# Analysis of complication management strategies after tympanoplasty under otoendoscopy

**DOI:** 10.3389/fsurg.2026.1847285

**Published:** 2026-06-09

**Authors:** Huping Huang, Xueyan Xie, Zhenhua Zhong, Kangsong Chen, Kai Sun, Junming Chen, Xiangling Jiang, Youjun Yu, Maohua Wang

**Affiliations:** 1 Department of Otolaryngology, Head and Neck Surgery, The First People's Hospital of Foshan, Hearing and Balance Medical Engineering Technology Center of Guangdong, Foshan, China; 2 Department of Otolaryngology, Head and Neck Surgery, The Affiliated Hospital of Yangzhou University, Yangzhou University, Yangzhou, China

**Keywords:** complications, endoscopic middle ear surgery, otoendoscopic surgery, treatment strategies, tympanoplasty

## Abstract

**Background:**

Complications from tympanoplasty not only impact surgical outcomes but also lead to severe patient suffering, with the absence of relevant literature on their treatment.

**Objectives:**

To summarize and explore the management strategies for complications after tympanoplasty under otoendoscopy.

**Materials and methods:**

A case series study was conducted on 770 patients (839 ears) who underwent otoendoscopic tympanoplasty at the First People's Hospital of Foshan between January 2024 and December 2024. Regular outpatient otoendoscopic follow-ups were performed after surgery, and the incidence of postoperative complications was recorded to investigate their causes, develop timely treatment strategies, and summarize the experiences gained.

**Results:**

Postoperative complications occurred in 142 ears, with an incidence of 16.9%. Specific complications included surgical area infection (17 ears), poor tympanic membrane healing (31 ears), myringitis (27 ears), exposed bone wall at the incision site (44 ears), granulation hyperplasia in the surgical area (20 ears), and stenosis of the external auditory canal (3 ears). All complications were managed using individualized treatment strategies.

**Conclusion and significance:**

Otoendoscopic tympanoplasty can lead to many complications. Regular outpatient follow-up is beneficial for the timely detection and management of complications, preventing serious consequences, improving surgical efficacy, reducing patient pain, and providing guidance to primary hospitals and young doctors.

## Introduction

Rehabilitation of conductive hearing loss resulting from chronic suppurative otitis media and cholesteatoma remains a significant challenge for otologists (1). Tympanoplasty is a classic surgical method for treating chronic middle ear diseases and reconstructing hearing. Its primary goal is to remove granulation tissue, cholesteatoma, obstructive cholesterol granulomas, and other pathological tissues in the middle ear (including the tympanic cavity and mastoid), while using temporal fascia, tragus cartilage membrane complex, and other materials to repair the tympanic membrane (2, 3). For many otologists, the occurrence and management of postoperative complications in tympanoplasty have long been a source of concern. Current research indicates that the complications of tympanoplasty mainly include surgical area infection, poor healing of the tympanic membrane, tympanitis, bone exposure at the incision site, granulation hyperplasia in the surgical area, re-perforation of the tympanic membrane, dizziness, hearing loss, narrowing of the external auditory canal, and implant retraction (4–7). These complications not only cause significant pain to patients but also affect the effectiveness of surgical treatment to varying degrees, with some patients even needing a second surgery.

Otoendoscopy is currently one of the most widely used tools for postoperative follow-up in otology. Combining different angles of otoendoscopy can help doctors comprehensively assess the healing of the surgical cavity and eardrum while also providing timely and effective management of postoperative complications to avoid serious consequences. This article summarizes the occurrence of complications after otoendoscopic tympanoplasty, analyzes and explores the causes, and develops treatment strategies to provide guidance for primary hospitals and young doctors.

## Materials and methods

### General information

The clinical data of 770 patients (839 ears in total) who underwent total ear endoscopic tympanoplasty at the First People's Hospital of Foshan between January 2024 and December 2024 were collected.

The inclusion criteria were as follows: (1) a confirmed diagnosis of chronic suppurative otitis media, middle ear cholesteatoma, adhesive otitis media, or ossicular chain discontinuity requiring endoscopic tympanoplasty (including secondary surgery); and (2) occurrence of postoperative complications during outpatient follow-up, including surgical area infection, delayed healing or inflammation of the tympanic membrane, exposed bone wall at the incision site, granulation tissue formation in the surgical area, and external auditory canal stenosis.

The exclusion criteria were as follows: patients who did not undergo otoendoscopic surgery, those who underwent conversion to a postauricular approach or microscopic-assisted procedure during surgery, and those who failed to complete regular postoperative follow-up visits.

A total of 142 ears were included in the study, with personalized treatment administered according to the specific complications observed in each patient. A comprehensive summary and analysis were conducted based on inpatient and outpatient medical records, surgical data, postoperative otoscopic reexamination images, and related documentation.

### Management strategies for postoperative complications

#### Surgical area infection

**Figure 1:**
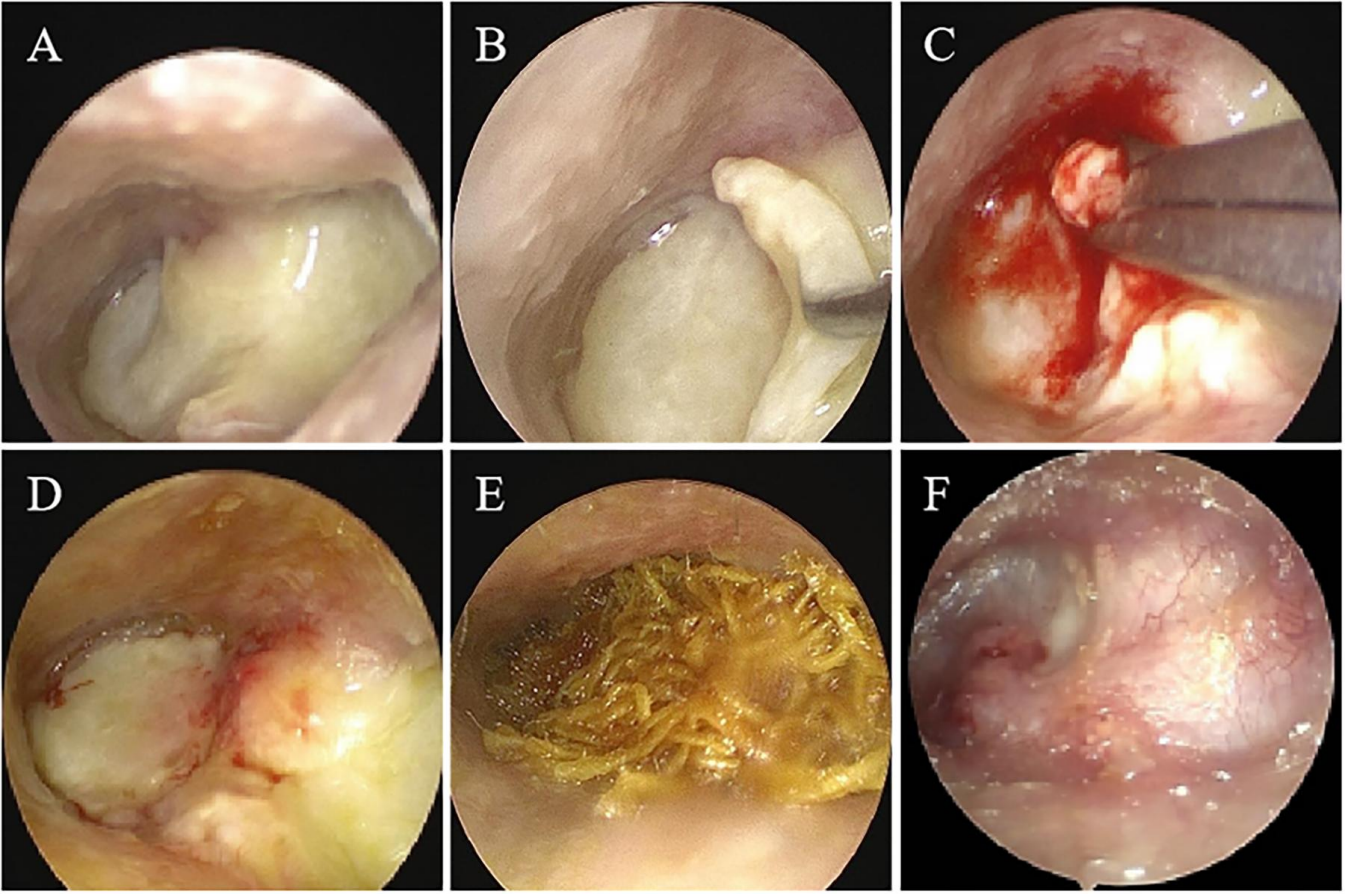
Management of postoperative infection. **(A)** Postoperative otoscopic examination revealed liquefaction of the gelatin sponge in the ear canal with visible purulent discharge. Following cleaning, poor vascularity of the flap was observed, accompanied by partial liquefaction and necrosis. **(B,C)** Debridement of liquefied and necrotic tissue under otoscopic guidance. **(D,E)** The ear canal was packed with an iodoform-impregnated gelatin sponge and iodoform gauze, with weekly dressing changes. **(F)** After 1 month of treatment, satisfactory epithelialization of the surgical cavity was achieved.

The predominant pathogenic organism in patients with surgical site infections is *Pseudomonas aeruginosa*. During dressing changes, dark-green secretions were frequently observed adhering to the packing material, accompanied by a characteristic foul odor. At this point, the gauze packing in the ear canal was promptly removed under otoscopic guidance. The gelatin sponge within the ear canal is often fully liquefied, and purulent exudate is evident upon examination. These secretions were collected for bacterial culture and antimicrobial susceptibility testing. The ear canal was then irrigated with chloramphenicol and dexamethasone solution. The surgical cavity was carefully inspected, and ischemic, liquefied, or necrotic tissues were debrided as necessary. Any floating fascia or subdermal secretions were gently expressed using a cotton applicator, avoiding direct suction to prevent tissue damage. The cavity was packed with a dexamethasone-soaked cotton ball; the use of a gelatin sponge is not recommended because of its tendency to rapidly liquefy. Empirical treatment with quinolone antibiotics is advised for infection control and may be adjusted based on the culture and sensitivity results. Concurrently, ofloxacin combined with dexamethasone otic drops was administered 2–3 times daily. Daily dressing changes are required until the surgical cavity is dry and clean, allowing ischemic floating tissues to gradually reattach and undergo epithelialization (Figure 1).

#### Poor healing of the tympanic membrane

**Figure 2:**
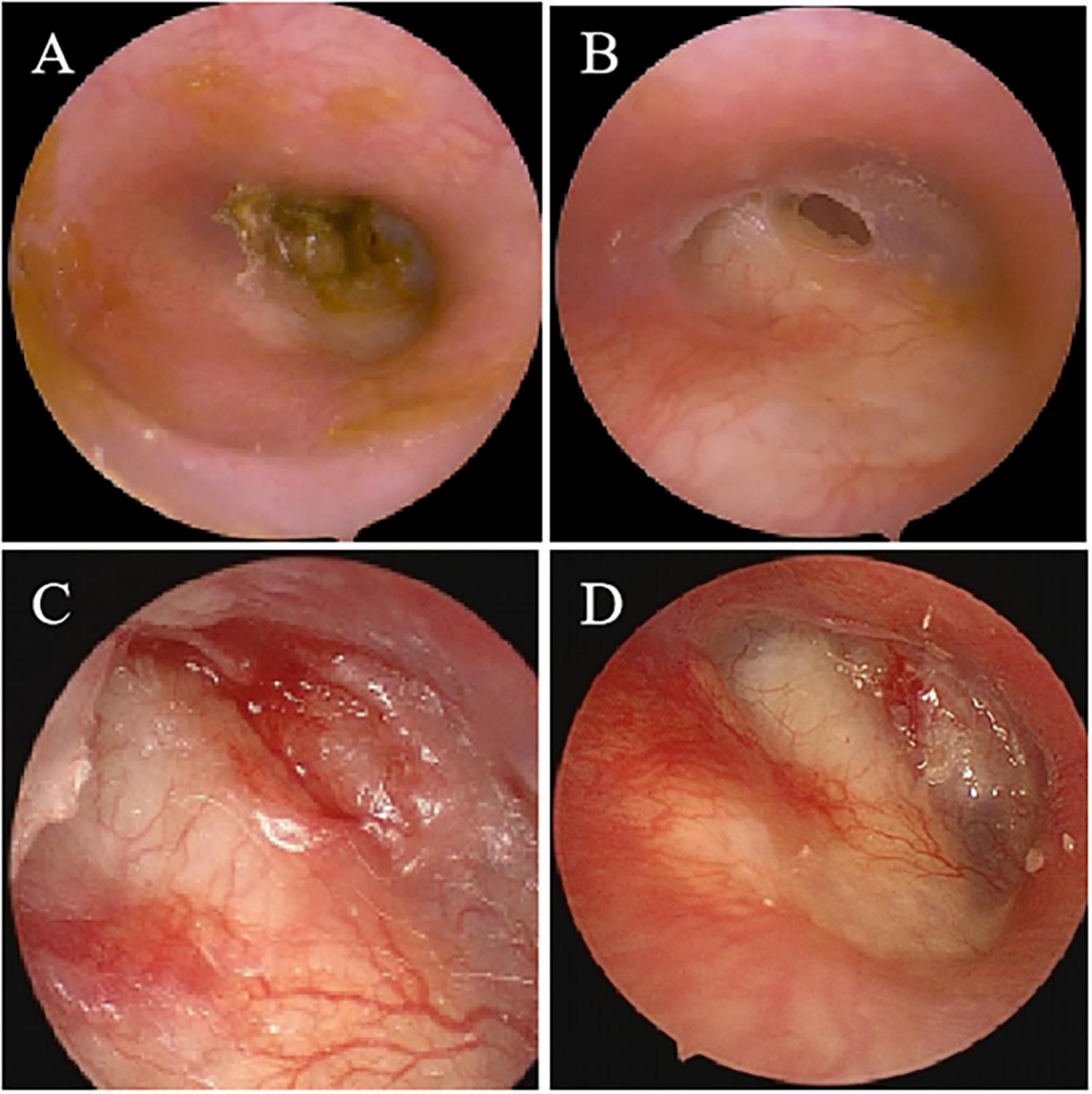
Postoperative management of tympanic membrane fissures. (**A**,**B**) A fissure in the anterosuperior portion of the tympanic membrane was observed during postoperative otoscopic examination. (**C**) The margins of the fissure were gently abraded under otoscopic guidance, followed by the application of an autologous dermal graft. (**D**) Complete healing was achieved within approximately 2 weeks.

**Figure 3:**
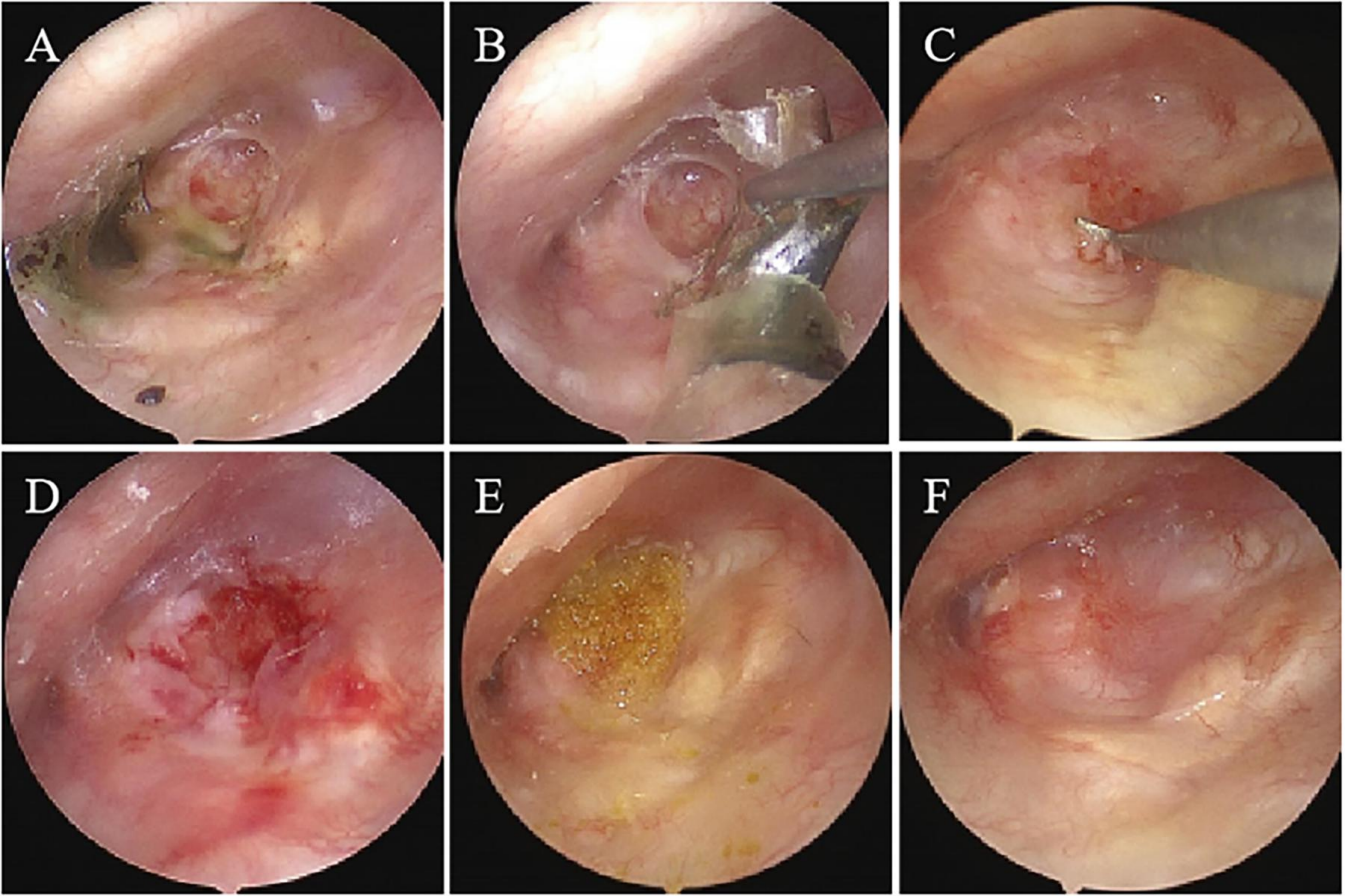
Postoperative management of tympanic membrane dissection. **(A,B)** Postoperative otoscopic examination revealed dissection between the residual tympanic membrane and the graft. **(C,D)** Under otoendoscopic guidance, the inner surface of the dissection was gently scraped, and radial incisions were made using a microscissor to facilitate close apposition of the graft. **(E)** The external cavity was filled with an appropriate amount of iodine-impregnated gelatin sponge, followed by gentle pressure application to promote adherence and healing. **(F)** The dissection healed within 2 weeks.

Poor healing of the tympanic membrane may manifest as the formation of interstitial layers, fissures, or even small perforations between the residual tympanic membrane and the graft. In cases of small perforations or fissures detected at an early stage, autologous fascia or dermal tissue can be applied via underlay grafting with the external placement of a dexamethasone-impregnated gelatin sponge to support healing. For small epithelialized tympanic membrane perforations, the margins should be gently curetted under otoendoscopy to create a fresh wound surface, thereby stimulating spontaneous healing. If healing does not occur, Allium sativum (garlic) skin grafts may be considered as an adjunctive patching material. In cases of separation between the residual tympanic membrane and the graft, after gentle abrasion of the inner surface under otoscopic visualization, radial incisions were made in the residual membrane at the site of separation using microscissors to facilitate apposition to the graft. Subsequently, a moderate amount of gelatin sponge was placed externally to apply gentle pressure and promote adherence. When a deep interlayer remains, and adequate apposition cannot be achieved after incision, fascia or dermis is inserted between the residual tympanic membrane and the graft to bridge the gap. This is followed by external packing with a gelatin sponge to ensure proper contact and healing (Figures 2 and 3).

#### Myringitis

**Figure 4:**
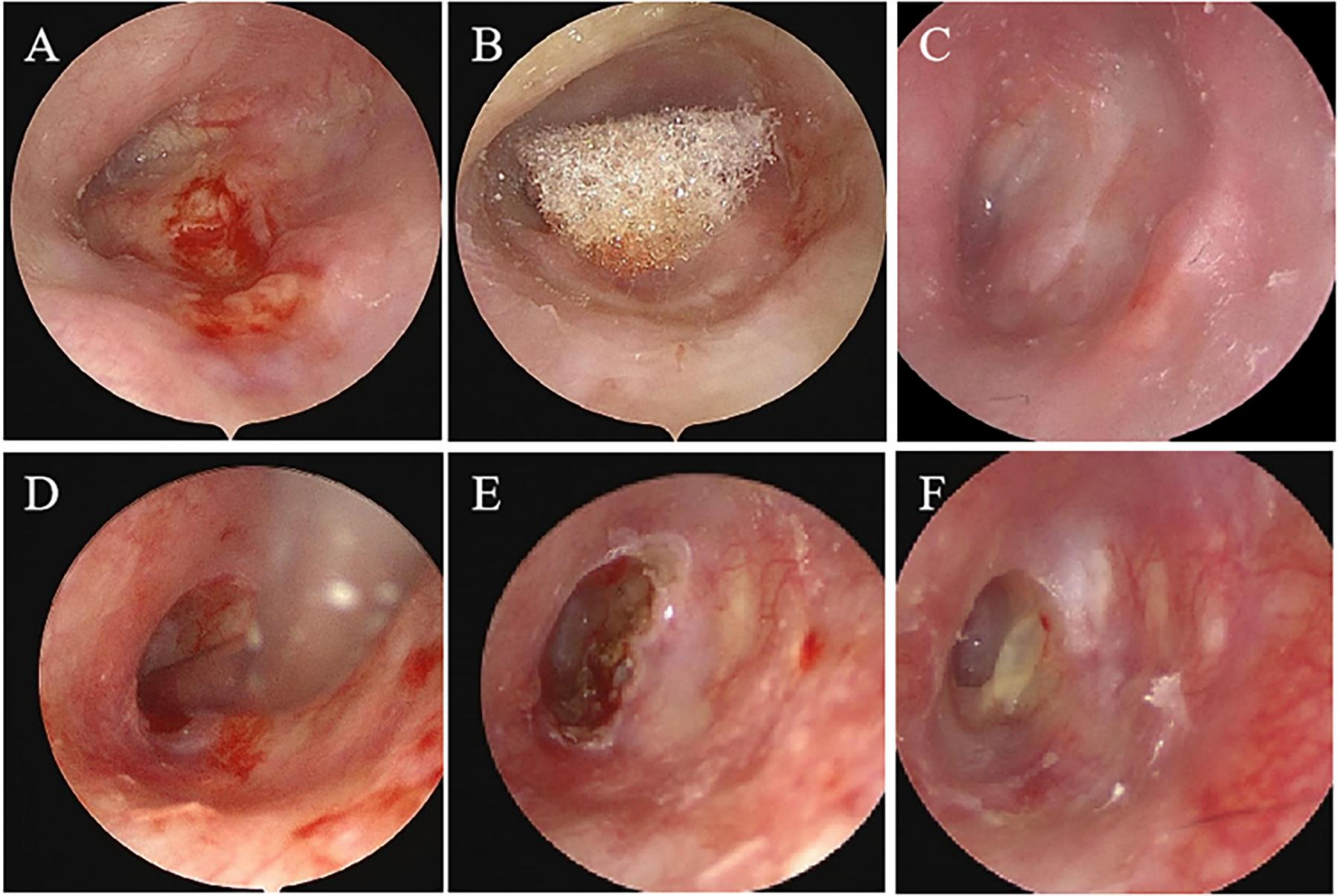
Management of postoperative myringitis. Panels **(A–C)** illustrate Case 1. Panel **(A)** shows postoperative otoscopic findings revealing the absence of epithelium in the posterior**–**superior region of the tympanic membrane and part of the posterior wall of the external auditory canal. Panel **(B)** demonstrates the targeted application of a dexamethasone-impregnated gelatin sponge via otoscopy to the denuded area. Panel **(C)** shows the resolution of tympanic membrane inflammation within 2 weeks. Panels **(D–F)** present Case 2. Panel **(D)** displays otoscopic evidence of epithelial loss in the posterior**–**superior quadrant of the tympanic membrane. Panel **(E)** illustrates bipolar cauterization of the affected area using otoendoscopy (Jesse technique), followed by the topical application of prednisone powder. Panel **(F)** shows near-complete resolution of inflammation at 2 weeks post-treatment.

This condition is commonly characterized by the absence of epithelium in tympanic membrane grafts or external auditory canal flaps, with mucopurulent secretions on the surface. It may be accompanied by granulation tissue formation and fungal colonization. During otoscopic examination, secretions and crusts within the ear canal were removed, and the denuded epithelial area was gently debrided with a hook needle. Following irrigation with dexamethasone solution, prednisone powder was topically applied to the affected surface. If granulation tissue is observed at the site of epithelial loss, it should be carefully curetted or excised. In cases of fungal infections, antifungal agents such as povidone-iodine or clotrimazole may be administered locally. For patients exhibiting persistent inflammation of the tympanic membrane despite repeated debridement and topical steroid application, radiofrequency ablation using a Jesse bipolar device may be considered an adjunctive treatment (Figure 4).

#### The bony wall at the incision site is exposed

**Figure 5:**
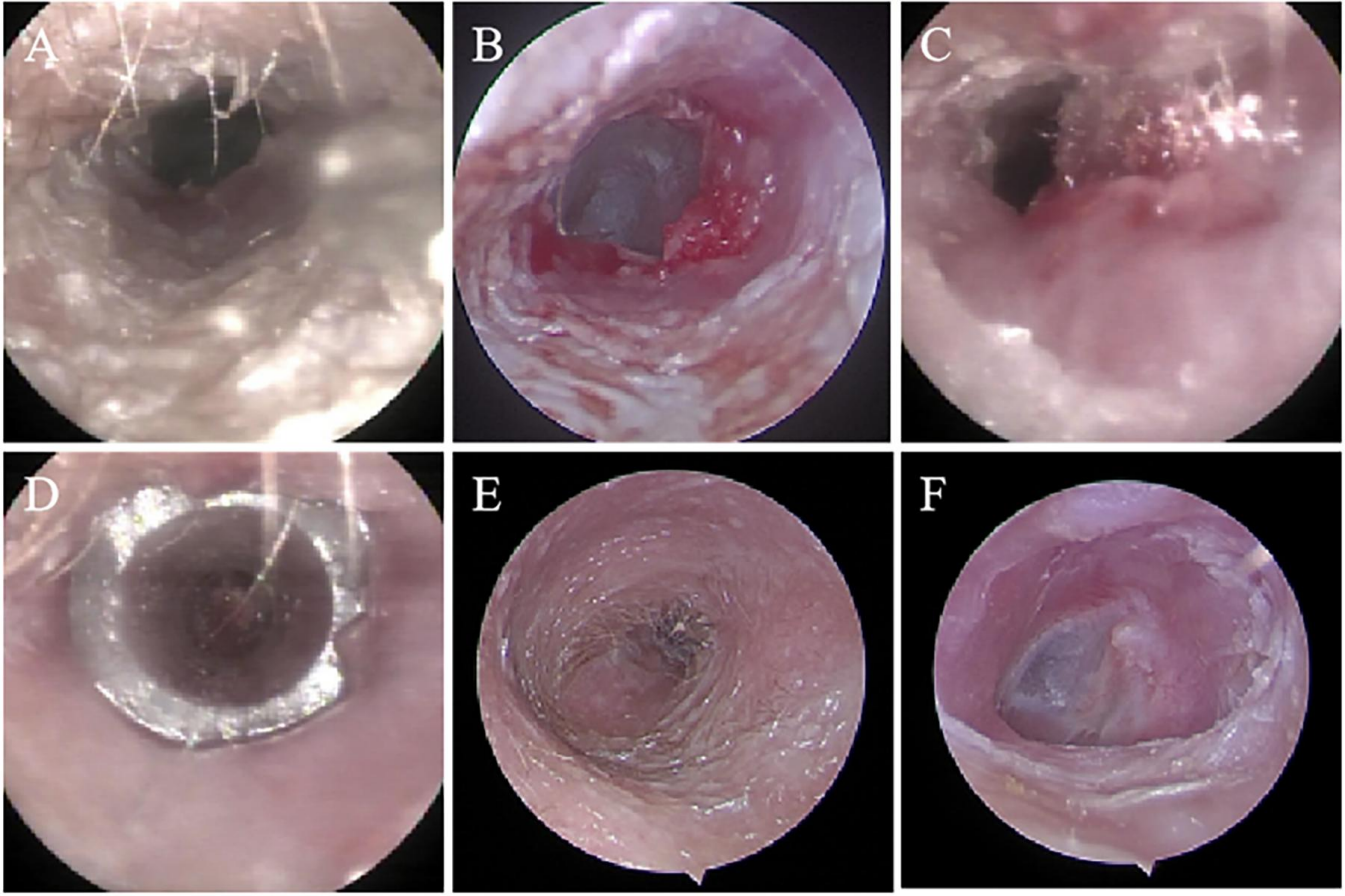
Management of the exposed bone wall at the surgical incision site. **(A,B)** Postoperative endoscopic examination revealed exposed bone at the incision site. **(C,D)** Under otoendoscopic guidance, the exposed bone surface and incision margins were debrided to create a fresh wound bed, followed by the topical application of dexamethasone combined with an erythromycin-impregnated gelatin sponge via wet compression. **(E,F)** After 2 weeks, the gelatin sponge was removed, and endoscopic evaluation confirmed complete wound healing.

This condition is characterized by incomplete healing at the incision site within the ear canal, resulting in exposure of the bony wall, which is frequently covered with thick crusts. During otoscopic examination, crusts were carefully removed using a curette, avoiding suction to prevent trauma. Following thorough cleansing of the ear canal, the exposed bone surface and margins of the flap were gently curetted until punctate bleeding occurred, thereby creating a fresh, viable wound bed. After blood aspiration, the area was covered with a gelatin sponge impregnated with dexamethasone and erythromycin ointment. The sponge should be kept adequately moist and replaced regularly, as premature desiccation may impair epithelial migration. Weekly follow-up is recommended to replace the sponge and assess the epithelialization progress. In cases where the exposed bone exhibits irregularities or compromised vascularity (evidenced by a pale, waxy appearance), localized debridement or grinding of the bone is indicated. For extensive areas of exposed bone, coverage with temporal fascia or dermal matrix graft may be necessary, followed by application of a dexamethasone-impregnated gelatin sponge over the surface (Figure 5).

#### Granulation hyperplasia in the surgical area

**Figure 6:**
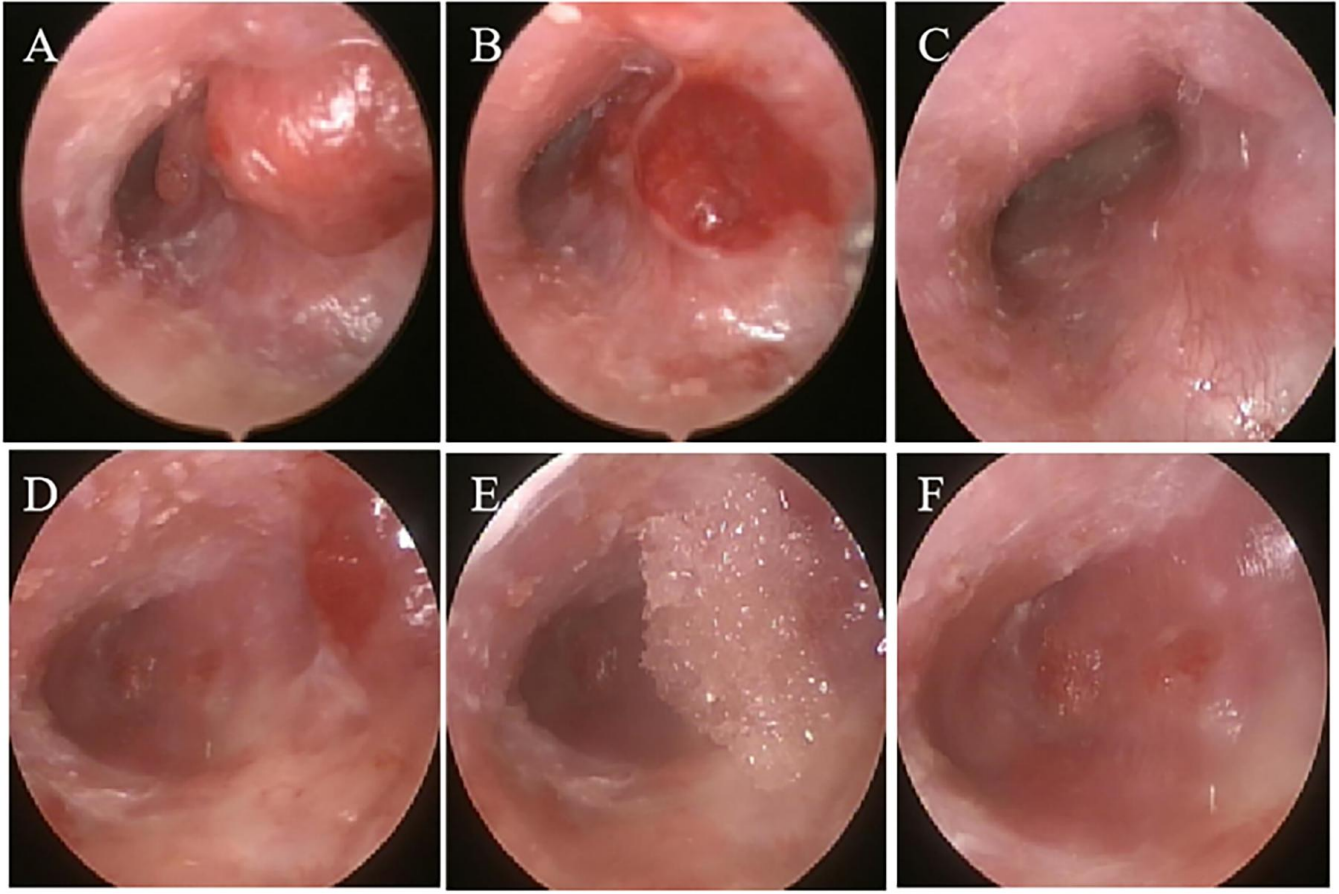
Management of granulation tissue hyperplasia at the postoperative incision sites. Panels **(A–C)** illustrate Case 1. **(A)** Otoscopic examination revealed significant granulation tissue hyperplasia at the incision site between the tragus and helix following surgery. **(B)** Under otoscopic guidance, the hyperplastic granulation tissue and its base were excised using forceps, followed by the topical application of prednisone powder to the affected area. **(C)** The wound exhibited favorable healing at 2 weeks post-intervention. Panels **(D–F)** present Case 2. **(D)** Postoperative otoscopy revealed granulation tissue hyperplasia at the surgical incision site. **(E)** After removal of the granulation tissue using forceps under otoscopic visualization, a dexamethasone-impregnated gelatin sponge was applied locally as a moist dressing. **(F)** Follow-up examination after 2 weeks demonstrated satisfactory wound healing.

It often manifests as granulation tissue hyperplasia at the incision site between the tragus and helix following radical mastoidectomy. This condition may lead to the liquefaction of the sponge material placed in this area, resulting in purulent discharge from the external auditory canal. In severe cases, it may result in ischemic necrosis of the fascia or dermal tissue covering the lateral aspect of the upper tympanic cavity and tympanomastoid recess. Under otoscopic guidance, the secretions in the ear canal were cleared, and the surgical cavity was irrigated. Granulation tissue was meticulously excised using microcup forceps until a smooth surface was achieved, followed by the topical application of prednisone powder. Patients were advised to attend weekly follow-up visits until complete wound healing was confirmed. For patients with limited access to follow-up care, dexamethasone-impregnated gelatin sponges may be applied to the wound surface as a wet dressing. Upon discharge, patients may self-administer the prescribed ofloxacin and dexamethasone otic drops once or twice daily until full resolution of the incision site (Figure 6).

#### Narrow external auditory canal

**Figure 7:**
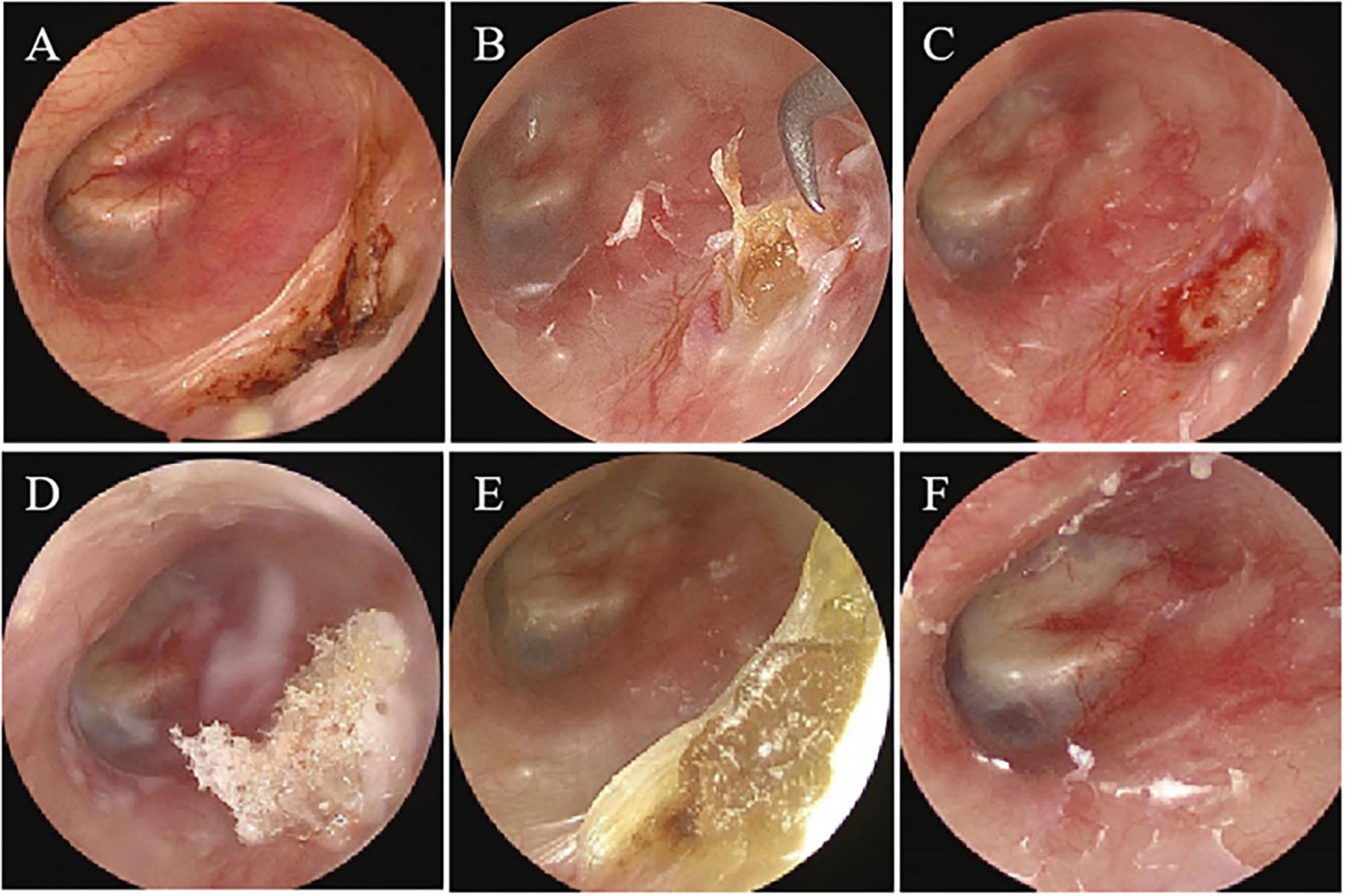
Management of postoperative external auditory canal stenosis. **(A)** Postoperative endoscopic examination revealed scar hyperplasia at the incision site and stenosis in the mid-portion of the external auditory canal. **(B)** Under otoscopic guidance, excessive hyperplastic scar tissue was partially excised. **(C,D)** Dexamethasone was locally injected into the stenotic site. A gelatin sponge was placed along the medial aspect of the ear canal to prevent direct contact between the silicone tube and the graft, thereby minimizing foreign body irritation that could induce granulation tissue formation on the graft surface. Subsequently, a silicone tube of appropriate size was inserted into the ear canal to maintain its patency and prevent restenosis. **(E,F)** At the 3-month follow-up, significant improvement in external auditory canal patency was observed, and the silicone tube was successfully removed.

It manifests as scar hyperplasia at the opening or incision site of the external auditory canal, often accompanied by auditory canal stenosis. Following otoscopic ear canal clearance, localized injection of dexamethasone is administered at the site of the most prominent scar formation—either weekly for 1 month or monthly thereafter. A silicone tube was subsequently placed to prevent restenosis. Prior to placement, an appropriate amount of gelatin sponge was inserted into the ear canal to avoid direct pressure of the tube on the graft, thereby minimizing the risk of granulation tissue formation on the tympanic membrane surface. The silicone tube should remain in place for 3–6 months (Figure 7).

## Results

### Basic information

In total, 839 ears from patients were monitored, and ultimately, 142 ears with postoperative complications were included in the study. This included 58 ears from male patients and 84 ears from female patients. The age range was 5–79 years, with a mean age of 45.63 ± 14.89 years. Among the cases, 75 ears had chronic suppurative otitis media, 59 ears had middle ear cholesteatoma, 3 ears had adhesive otitis media, and 5 ears had ossicular chain discontinuity.

### The incidence and management strategies of complications

Patients were followed up postoperatively until the surgical site was dry and clean, the tympanic membrane perforation had healed, complete epithelialization of the surgical site was achieved, or until 6 months after surgery, whichever occurred first.

Postoperative infections occurred in 17 ears, including two with tragal incision site infections and 15 with surgical area infections, of which four were confirmed to be fungal infections. All infected cases underwent thorough debridement using otoendoscopy. Necrotic tissue was removed, secretions were collected for microbial culture, and targeted antibiotic therapy was administered based on the culture results. A dry ear was achieved in all patients within 2–3 weeks, and no tympanic membrane re-perforation was observed in any patient. Five patients with postoperative infection had secondary operative site bone exposure. After a comprehensive analysis, we confirmed that bone exposure was a secondary change caused by postoperative infection rather than an independent concurrent complication. Tympanic membrane fissures were observed in 19 ears. After otoendoscopic patching with fascia or dermal matrix, 18 ears healed within 1 week, while a small residual perforation was observed in 1 ear at 2 weeks post-treatment. After scraping and garlic skin patching, it healed after 2 weeks. A separation between the residual tympanic membrane and the graft was observed in 13 ears. The residual tympanic membrane was radially cut open to tightly adhere to the graft under otoendoscopy, and the outer side was filled with a gelatin sponge. The interlayer healed within 1–2 weeks. Postoperative tympanic membrane inflammation was observed in 27 ears. The scab and secretions on the surface of the tympanic membrane were cleaned under otoendoscopy, a crochet hook was used to scratch the epithelial deficiency area to create a fresh wound, and prednisone powder was sprayed simultaneously. Of these, 22 ears had complete epithelialization within 1–2weeks. In the remaining five cases, limited improvement was observed, followed by complete epithelialization within 3–4weeks after radiofrequency treatment using Jesse Bipolar. Non-healing incisions with exposed bone walls were observed in 44 ears. After scraping and covering with dexamethasone and erythromycin gelatin sponge under otoendoscopy, 35 ears healed within 4 weeks. Nine ears still had exposed bone walls 3 months after surgery, of which seven ears were covered with a gelatin sponge at the incision site and self-dripped with ofloxacin and dexamethasone ear drops. By the 6-month follow-up after surgery, they had already healed. Two additional ears had large defect areas and were repaired with fascia or a dermal matrix. In 32 ears, granulation hyperplasia was observed in the surgical area. After trimming the granulation tissue under otoendoscopy and the application of prednisone powder, 28 ears achieved complete epithelialization within 8 weeks. Incomplete epithelialization persisted after the 3-month follow-up in four ears. The treatment involved using wet compresses with a dexamethasone-impregnated gelatin sponge, which was applied one to two times until the condition resolved. External auditory canal stenosis was diagnosed in three ears, all attributed to scar hyperplasia at the incision site. Under otoendoscopy, 1 mL of dexamethasone was injected weekly into the stenotic segment for 1 month. A silicone tube was concurrently inserted to prevent restenosis. By following this combined approach, a significant improvement in canal patency was observed, with no recurrence of stenosis during the 6-month follow-up period (Table 1).

**Table 1:** Incidence and management strategies of complications.

Complication	No. of cases	Management strategy	Clinical outcomes
Surgical area infection	17	1. All infected cases underwent thorough debridement under otoendoscopy 2. Necrotic tissue was removed, secretions were collected for microbial culture, and targeted antibiotic therapy was administered based on culture results	All cases were dry and completely epithelialized
Poor healing of the tympanic membrane	31	1. Using fascia or dermal matrix for patching under otoendoscopy 2. Scraping and garlic skin patching 3. The residual tympanic membrane was radially cut open to tightly adhere to the graft under otoendoscopy, and the outer side was filled with gelatin sponge	All perforations and dissections were healed
Myringitis	27	1. Cleaned the scab and secretions on the surface of the tympanic membrane under otoendoscopy, used a crochet hook to scratch the epithelial deficiency area to create a fresh wound, and sprayed prednisone powder 2. Radiofrequency treatment using Jesse bipolar	All cases were completely epithelialized
The bony wall at the incision site was exposed	44	1. Scraped and covered with dexamethasone and an erythromycin gelatin sponge under otoendoscopy 2. Covered with gelatin sponge at the incision site and self-dripped with ofloxacin and dexamethasone ear drops 3. Repaired with fascia or a dermal matrix	All cases were completely epithelialized
Granulation hyperplasia in the surgical area	20	1. Trimmed the granulation tissue under otoendoscopy and sprayed prednisone powder 2. Managed with wet compresses using a dexamethasone-impregnated gelatin sponge	All patients had smooth surgical cavities and complete epithelialization
Narrow external auditory canal	3	1. 1 mL of dexamethasone was injected weekly at the stenotic segment for a month 2. A silicone tube was inserted to prevent restenosis	All patients showed no progression of external auditory canal stenosis

## Discussion

Tympanoplasty is a widely used surgical intervention for the management of chronic otitis media, cholesteatoma, and ossicular chain discontinuity. It encompasses various surgical techniques. Regardless of the specific approach employed, postoperative complications such as cavity infection, tympanic membrane graft failure, tympanosclerosis, acquired external auditory canal stenosis, and lesion recurrence have persistently presented clinical challenges for both patients and otologists (8–10). As reported by Wang et al., suboptimal recovery following totally endoscopic middle ear surgery mainly manifests as poor tympanic membrane healing, recurrent retraction adhesion, unimproved or aggravated hearing, and cicatricial canal atresia. This highlights the importance of regular follow-up for early detection and intervention (11). Regular postoperative outpatient follow-up via otoendoscopy enables the early identification of these complications, facilitating timely intervention to prevent adverse outcomes, enhance surgical success rates, and promote accelerated patient recovery.

Otoendoscopy provides several advantages, such as 4 K high-definition imaging, close-up visualization, and a wide-angle field of view, which facilitate the detection of lesions and subtle pathological changes in anatomically concealed areas, thereby enabling timely and targeted therapeutic interventions for patients (12–15). Outpatient otoscopic follow-up allows otologists to perform direct visualization-guided clearance of external auditory canal obstructions, secretions, and necrotic tissue debridement with enhanced precision, thereby minimizing trauma to the surrounding healthy tissues. Our department was the first to develop otoendoscopic technology in China. Over the past two decades, we have systematically integrated this technique into routine outpatient examinations, preoperative assessments, and therapeutic procedures. We have continuously expanded its surgical applications while accumulating extensive clinical experience in managing postoperative complications associated with otoendoscopic interventions.

Postoperative infection is one of the most prevalent complications following tympanoplasty, primarily attributable to bacterial or fungal pathogens. These infections may manifest as tragal incision site involvement or extend into the surgical cavity. Several predisposing factors have been identified, including compromised host immunity, inadequate preoperative control of active infection, deviations from strict aseptic techniques during surgery, incomplete preoperative removal of diseased tissue, inappropriate postoperative antibiotic administration, and non-standardized wound dressing procedures. Effective management of postoperative infection centers on four key principles: controlling microbial proliferation, eliminating the infectious source, preserving graft integrity, and preventing re-perforation of the tympanic membrane (16). Most of such infections occur within the first 1–2 weeks postoperatively. During otoscopic follow-up, it is essential to adhere to core principles that ensure a dry and clean surgical cavity, facilitate optimal wound healing, and minimize the risk of infection. Although transient moisture in the surgical cavity may provide short-term protection to the nascent epithelium, prolonged dampness creates a favorable environment for bacterial and fungal growth. This risk is particularly heightened in the presence of retained exudate or residual foreign material, which significantly increases the likelihood of infection (17, 18).

Poor healing of the tympanic membrane following surgical intervention is primarily characterized by necrosis, displacement, residual perforation, retraction, adhesion, and interlayer graft healing. The occurrence of these complications is predominantly associated with multiple factors, including surgical technique, disruption of the local microenvironment, and irritation from packing materials (5, 10). This study mainly focused on postoperative fissuring and dissection of the tympanic membrane. Potential contributing factors include inadequate packing in the anterior tympanic cavity and around the eustachian tube orifice during surgery, excessive dissection of the external auditory canal flap, persistence of blood or air bubbles beneath the graft, leading to incomplete apposition between the graft and remnant tympanic membrane, insufficient graft size, graft atrophy or displacement, persistent negative pressure within the middle ear, and compromised postoperative vascular supply to the graft. To mitigate these risks, surgeons should harvest grafts with adequate dimensions to ensure sufficient coverage. During tympanic cavity packing, priority should be given to securing proper support in the anterior region and at the eustachian tube orifice. Prior to packing the external auditory canal, careful inspection must be performed to ensure complete adherence between the residual tympanic membrane and the graft. If adherence is suboptimal, radial incisions in the residual membrane or the placement of supplemental fascia may be indicated based on clinical judgment. Cartilage reinforcement is recommended for structural stability in cases of an open tympanic cavity. Local trauma to the tympanic membrane resulting from ear surgeries is one of the primary etiological factors of tympanic membrane inflammation. Both intraoperative manipulation and excessive postoperative cleaning may contribute to the development of these conditions. The underlying pathogenesis of postoperative tympanic membrane inflammation involves injury to the epithelial layer of the tympanic membrane, in conjunction with chronic inflammatory stimulation (19). During surgical procedures, every effort should be made to minimize instrument-induced damage to the tympanic membrane epithelium. In the postoperative phase, otoscopic cleaning should follow the principle of “moderate debridement”. It is important to avoid aggressive removal of cellulose membranes or newly formed epithelium on the wound surface, as such practices may compromise the natural repair barrier of the tympanic membrane and increase the risk of postoperative myringitis (20). Postoperative bone exposure at the surgical site is primarily associated with compromised healing of the ear canal flap and inadequate vascular supply to the surrounding bone, often due to intraoperative factors. Excessive contraction of the external auditory canal flap may lead to extensive exposure of the bony wall during final closure. The use of energy-based instruments, such as monopolar electrocautery or plasma knife tips, during incision creation poses a risk if excessive energy levels or prolonged contact times are employed, potentially impairing local perfusion to the flap or underlying bone and thereby predisposing to postoperative bone exposure. Furthermore, over-dissection of the flap or aggressive undermining of the anterior canal skin can disrupt critical blood supply routes, leading to flap ischemia and subsequent bone exposure. In procedures involving an open tympanomastoid cavity, insufficient flap size, excessive tension, or poor apposition between the flap and bone surface may prevent secure adherence, increasing the likelihood of flap “floating,” which can progress to ischemic necrosis and exposed bone. Postoperative granulation tissue hyperplasia at the surgical site is primarily attributed to an excessive reparative response of local tissues to injury, inflammation, or the presence of foreign bodies. This condition may be associated with excessive traction or trauma to the flap during surgery, inadequate adhesion of the graft to the ear canal wall leading to postoperative displacement and subsequent irritation of surrounding tissues, as well as chronic inflammatory stimulation resulting from postoperative infection. To reduce the incidence of postoperative granulation tissue proliferation, when making an incision in the ear canal during surgery, the cutting edge should be kept as neat as possible along the same direction. When peeling off the skin flap, gentle movements should be made to maintain the integrity of each layer of the flap as much as possible. Before filling, it should be ensured that the skin cutting edge is tightly adhered to the bone wall, and the residual edge of the tympanic membrane is tightly adhered to the graft. When filling, a gelatin sponge coated with erythromycin ointment can be applied to the surface of the flap incision to promote wound healing and prevent granulation growth.

In summary, we have, for the first time in China, conducted a comprehensive analysis of clinical data from patients experiencing postoperative complications after otoendoscopic tympanoplasty. We have thoroughly explored the causes of each complication in detail, identified key precautions for preoperative and intraoperative care, and developed a complete set of management measures for postoperative complications. We are able to deal with each complication in a timely and effective manner, and our quantity and efficacy are in a leading position in China. This provides guidance for most physicians engaged in otosurgery, greatly shortens the growth cycle of grassroot and young doctors, reduces the pain of patients, and accelerates postoperative recovery.

However, the limitations of this study include the case series study design, potential selection bias caused by the exclusive inclusion of complication cases, the absence of a control group, and the inevitable operational variability among different surgeons. Therefore, there was a certain bias in the incidence of postoperative complications. We hope to address these limitations in future studies.

## Data Availability

The original contributions presented in this study are included in the article/Supplementary Material. Further inquiries can be directed to the corresponding author/s.
